# Wing Design in Flies: Properties and Aerodynamic Function

**DOI:** 10.3390/insects11080466

**Published:** 2020-07-23

**Authors:** Swathi Krishna, Moonsung Cho, Henja-Niniane Wehmann, Thomas Engels, Fritz-Olaf Lehmann

**Affiliations:** Department of Animal Physiology, Institute of Biosciences, University of Rostock, 18059 Rostock, Germany; swathi.krishna@uni-rostock.de (S.K.); moonsung.cho@uni-rostock.de (M.C.); henja-niniane.wehmann@uni-rostock.de (H.-N.W.); thomas.engels@uni-rostock.de (T.E.)

**Keywords:** locomotion, animal flight, wing structure, aerodynamics, flight force

## Abstract

The shape and function of insect wings tremendously vary between insect species. This review is engaged in how wing design determines the aerodynamic mechanisms with which wings produce an air momentum for body weight support and flight control. We work out the tradeoffs associated with aerodynamic key parameters such as vortex development and lift production, and link the various components of wing structure to flight power requirements and propulsion efficiency. A comparison between rectangular, ideal-shaped and natural-shaped wings shows the benefits and detriments of various wing shapes for gliding and flapping flight. The review expands on the function of three-dimensional wing structure, on the specific role of wing corrugation for vortex trapping and lift enhancement, and on the aerodynamic significance of wing flexibility for flight and body posture control. The presented comparison is mainly concerned with wings of flies because these animals serve as model systems for both sensorimotor integration and aerial propulsion in several areas of biology and engineering.

## 1. Introduction

Insect wings are complex, three-dimensional structures that are under selective pressures towards functional optima. These optima result from multiple requirements, and also from evolutionary influences relevant to the animal’s fitness. Wings have mainly evolved for locomotion and produce aerodynamic forces during gliding and flapping flight at high wing beat frequencies of up to 1000 Hz [1]. The air flows generated for flight mainly depend on wing kinematics, the wing’s overall planform, and the dynamics of elastic deformation owing to inertial and aerodynamic loading. Pinpointing the factors that shape the evolution of wings and flapping kinematics is key to any in-depth understanding of flight. Within the past decades, numerous comprehensive reviews and book chapters have been published on insect flight, focusing on components such as aerodynamic mechanisms for lift enhancement [2,3,4,5,6,7,8,9,10,11], power requirements for wing flapping [12,13,14,15], wing kinematics and control [16,17,18,19,20,21], and the efficiency with which muscle mechanical power is turned into weight supporting lift [22,23]. This review is engaged in the link between three-dimensional wing structure and aerodynamics, focusing on recently published studies on the aerodynamic performance of wings in differently-sized insects. The review highlights the behavior of wings in flies because these animals often serve as model systems for aerial propulsion in both biology and engineering.

Insect wings receive their mechanical strength and endurance from two main components: on the microscopic level, the three-dimensional composition of proteins and chitin-based cuticle layers [24,25,26,27], and on the macroscopic level, the distribution and three-dimensional morphology of veins and elastic interconnecting membranes [28,29,30,31,32,33]. This light-weight design helps insect wings to widely resist external forces using chitin as the main chemical component [34]. Veins greatly vary in density, size, and shape between animal species and determine the wing’s structure and mechanical behaviors under load, such as bending and twisting [29,35,36,37,38,39]. Veins provide structural support to a wing, preventing the wing from tear [40,41] and host sensory receptors such as campaniform sensilla and innervated bristles, including their afferent nerves [42,43,44,45,46,47]. By contrast, wing membranes are aerodynamic active surfaces and composed of multiple layers of cuticle [25,27,48] with a thickness ranging from ~0.5 µm in small insects to ~1.0 mm in forewings (elytra) of large beetles [28,49]. Veins and membranes form fine geometrical structures that are typically of much smaller scale than the primary flow structures at wings, such as wing tip and leading edge vortices, and referenced as wing corrugation [50]. Coarse-scale structures, by contrast, typically refer to the wing’s overall curvature and termed chordwise and spanwise wing camber [51]. Throughout the past decades, several technical developments, such as high-resolution micro-computed tomography (μCT), have helped to better understand the various aspects of wing morphology for structural integrity [27,52], while robotic and numerical studies on insect flight have highlighted the aerodynamic significance of three-dimensional wing design [53,54,55,56,57].

Numerous studies have been published on the aerodynamic performance of translating [58,59,60,61,62,63,64,65,66] and root-flapping rigid wings [8,67,68,69,70,71,72,73,74]. The aerodynamics of dynamically deforming insect wings, by contrast, is less clear. Wing bending and twisting change the wing’s local angle of attack during flapping motion. Wing bending and twist is thus similar to changes in wing kinematics and change flow and force production. Wings may have an anisotropy in mean stiffness for ventral versus dorsal loading that unbalances force production during upstroke and downstroke, even in cases in which wing hinge articulation is the same in both halfstrokes [27,37,75]. Moreover, as spanwise stiffness in insect wings is approximately one to two orders of magnitude larger than chordwise stiffness, wings often deform in a characteristic fashion [37,76]. There is a continuing debate on the potential benefits of dynamic shape changes in flapping flight because some authors reported aerodynamic advantages of wing deformation for lift production [77,78,79,80], while other authors found disadvantages [80,81,82].

In this review, we work out the significance and tradeoffs of wing design for aerodynamic key parameters such as vortex development and lift production. This is achieved by disassembling the wing’s various properties and linking the components in wing structure to aerodynamics, power consumption and flight efficiency. The sections start with flow phenomena in a simple, flat, rectangular wing. In the second section, we focus on the benefits of elliptical and tapered wing shapes as found in many species, including flies. This section also highlights that even simple genetic modifications of fly wing planforms lead to measurable changes in aerodynamic performance. In the third section, we consider the wing’s three-dimensional morphology. A recent numerical study, for example, showed that the three-dimensional shape of rigid fly wings attenuates both lift production and aerodynamic efficiency rather than enhancing these measures compared to a flat wing [83]. In the last section, we focus on the aerodynamic consequences of elastic deformation in morphological complex wings. Although elastic wings share similar fluid dynamic properties with rigid wing, an animal must cope with the dynamically changing conditions because these changes may attenuate the ability and precision of flight and body posture control.

## 2. Aerodynamic Properties of Root-Flapping Rectangular Wings

**Figure 1:**
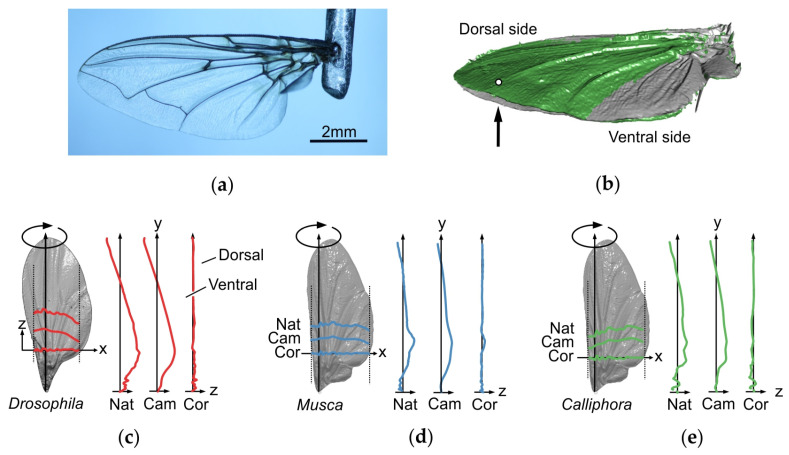
Characteristics of fly wings. (**a**) Detached wing of the blowfly *Calliphora vomitoria*, mounted to a steel holder. (**b**) Deformation of a blowfly wing (green) during loading by a ~64 µN point force (white dot) applied normal to the ventral wing side (arrow) [75]. Grey, surface profile without load. (**c**–**e**) Spanwise and chordwise wing profiles along the axes of rotation in three differently-sized fly species (*Drosophila melanogaster*, *Musca domestica*, *Calliphora vomitoria*). The wing profiles are superimposed on natural wing models (grey). The profiles separately show wing camber (Cam) and wing corrugation (Cor). Both wing components were numerically extracted from the natural wing shape (Nat) according to a procedure outlined in Engels et al. [83]. The out-of-plane component (z) is exaggerated by a factor of 2 for better clarity.

**Figure 2:**
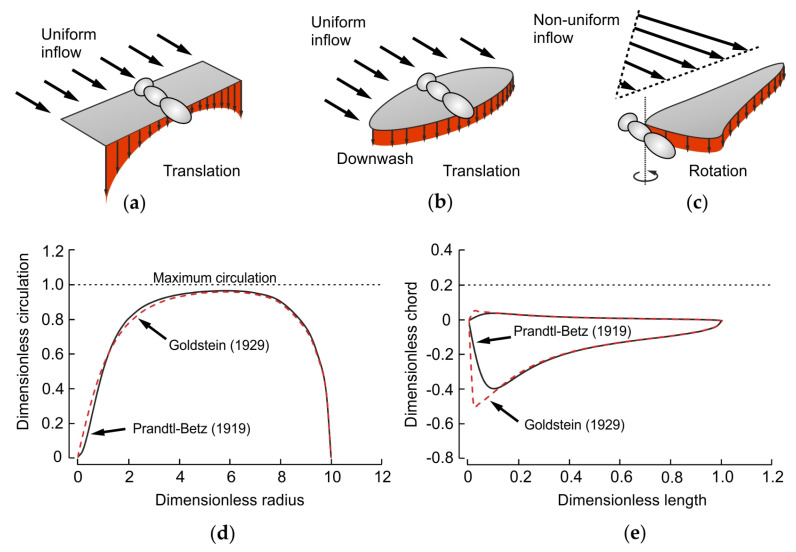
Ideal distribution of spanwise lift in translating and revolving wings. Distribution of vertical downwash velocity during translation in an (**a**) rectangular and (**b**) elliptical insect wing. At constant forward flight velocity, the inflow towards the wing is uniform. The ideal elliptical wing shape spreads spanwise vorticity that produces maximum span and Rankine–Froude efficiencies. (**c**) In a revolving wing, the non-uniform inflow requires adjustments in wing shape for maximum efficiency. (**d**) Distribution of spanwise circulation in an elliptical wing according to Prandtl [109], Betz [110] and Goldstein [111]. (**e**) Ideal wing shape for maximum span efficiency in a revolving wing according to Prandtl–Betz and Goldstein (see Supplementary Materials).

Rigid, flat, rectangular wings are often used to understand fundamental aerodynamic principles and represent the most simple approach towards insect flight [84] (Figure 1). They are investigated at different kinematic patterns such as revolving [85,86,87] and pitching motions [88,89,90,91]. Most studies though focused on the dynamics of the leading edge vortex that develops on the upper wing side at high angle of attack [8,72,92,93,94,95,96,97,98,99,100]. In contrast to a translating wing at high Reynolds number, the leading edge vortex in root-flapping and revolving insect wings is stably attached to the dorsal wing surface and enhances lift throughout the stroke cycle [72,92,101]. It obtains its stability from the viscosity of air and axial flow between wing hinge and wing tip [102,103]. Although a rectangular root-flapping plate produces all characteristic types of vortices and flows typical for insect wings, it suffers from low span efficiency compared to an elliptically shaped insect wing. Span efficiency is similar to Rankine–Froude efficiency, which typically refers to mean efficiency of propulsion in a complete wing flapping cycle of an animal during hovering conditions [104,105]. By contrast, instantaneous span efficiency varies during wing flapping and is the ratio between ideal power requirements for lift production and the actual requirements [106]. Span efficiency is maximum when the distribution of vertical velocities is uniform in the wing’s downwash [107,108]. Under this condition, the kinetic energy of the downwash is minimal owing to the non-linear, velocity-squared relationship between kinetic energy and wake velocity. If velocities vary within the wake, the velocity-squared relationship produces costs at elevated velocities that are not saved by the regions with low fluid velocities (Figure 2).

A pair of translating, flat wings has maximum span efficiency if it produces an elliptical lift distribution from tip to tip (Figure 2b) [109]. Span efficiency depends on the geometry of a wing, i.e., planform and camber, and its kinematics, but not on the wing’s aspect ratio and wing loading [107]. In general, the left and right wing of a two-winged insect can either be considered a single aerodynamic system or both wings may function as two aerodynamically independent systems. In the first case, each wing should have a semi-elliptical shape that results in an ellipse if both wings are connected via the insect body, where as in the second case each wing should have an elliptical shape for maximum span efficiency. Both geometrical cases yield higher span efficiency than a translating rectangular wing with same aspect ratio, and are thus beneficial for gliding flight of an insect. However, this conclusion only holds if the wings are flat and not twisted because an appropriate twist of a rectangular wing may equalize the downwash distribution via changes in local angle of attack.

**Figure 3:**
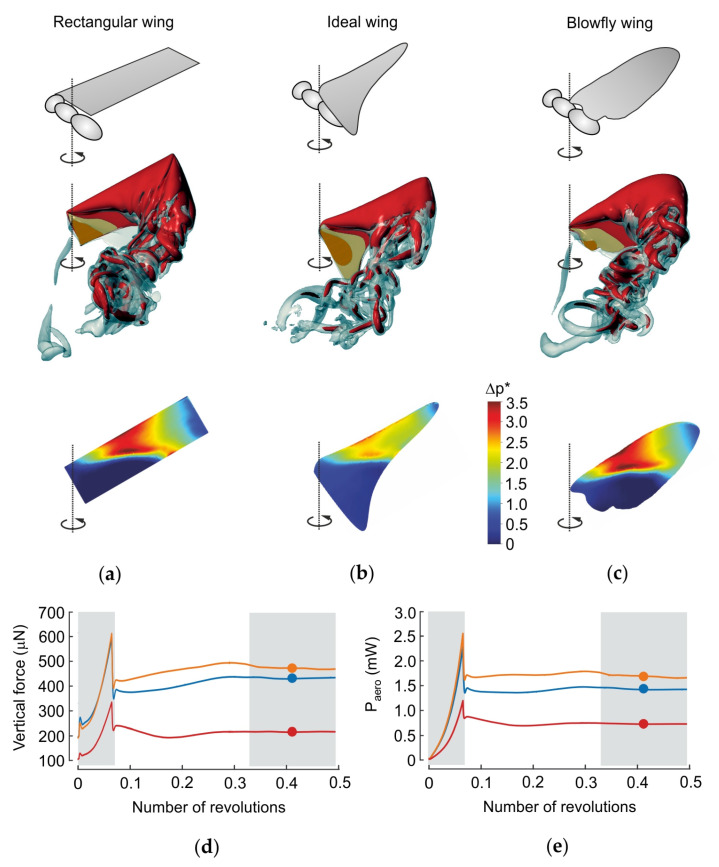
Aerodynamics of revolving wings. (**a**–**c**) Upper row: aerodynamic characteristics of three flat, continuously revolving wings (rectangular wing, ideal wing for rotation, wing of a blowfly). Middle row: data show iso-surface with vorticity magnitude of 75 s^−1^ (grey) superimposed on a vorticity iso-surface with 150 s^−1^ (red). The flow is shown after ~0.4 revolutions after motion onset. Lower row: pressure difference (Δp *) between dorsal and ventral wing sides, and normalized to the uniform wing loading pressure. The latter value is equal to body weight divided by the surface area of two wings. (**d**,**e**) Time evolution of vertical lift in *d* and aerodynamic power in *e*. After motion onset (grey, left), lift and power stabilize approximately after 0.3 revolutions (grey, right). Dots are mean values calculated from ~0.32–~0.5 revolutions (grey, right). Wing length and area are identical in all wings. For numerical modeling see [83]. Orange, rectangular wing; blue, wing of *Calliphora vomitoria*; and red, ideal-shaped wing.

In contrast to translating wings, in revolving and root-flapping wings, local blade velocity increases with increasing distance from root to tip, producing a non-uniform inflow distribution (Figure 2c). This changes the ideal, root-to-tip elliptical distribution in circulation (Figure 2d). Thus, an elliptical wing does not produce a uniform downwash distribution during revolving or root-flapping motion, requiring an eccentric planform for maximum span efficiency. Betz, Prandtl and Goldstein [110,111,112] estimated the optimal distribution of circulation in flat propeller wings, assuming flow leakages at the tip and root and thus zero circulation at the revolving axis (Figure 2c). Based on their results, we estimated the optimal wing shape in Figure 2e and for the calculations in Figure 3 (see Supplementary Materials). In contrast to Betz and Prandtl, Nabawy and Crowther [113,114,115] derived the optimal wing shape of two revolving wings assuming the elliptical circulation distribution of a pair of translating wings, with maximum circulation at the revolving axis. In this theoretical case, wing chord must continuously increase from wing tip to root in order to compensate for the drop in inflow velocity, leading to an “optimum” wing shape [114,115]. However, the latter design cannot produce a uniform downwash as in Prandtl–Betz’s estimate. In sum, the expected lower span efficiency in a rectangular wing may have fueled the evolution of elliptical insect wings for gliding flight. The expected lower span efficiency of elliptical wings during wing flapping, by contrast, might have led to the development of wing shapes that taper off towards the wing tip. Besides numerous biological pressures on wing planform development, it should be noted that span efficiency is only one aerodynamic factor that determines the costs of wing flapping as other costs such as inertial power requirements may also significantly contribute to total flight power expenditures [116].

## 3. The Aerodynamic Benefits of an Ideal Planform

Wing shape in insects is diverse. Significant shape measures are aspect ratio and the wing’s planform. High aspect ratio wings minimize induced drag and provide high lift-to-drag ratios by reducing the three-dimensional flow effects associated with tip vortices [117]. Aspect ratio also determines the stability of the leading edge vortex during wing flapping [117]. There is a wide variety of aspect ratios found in insect wings ranging from approximately 1.5 to 5.8 [118,119,120,121,122]. In Diptera, previous studies reported aspect ratios of 2.91–3.14 for *Drosophila* [121,122], 2.88 for *Musca* [83], and 2.62–2.93 for *Calliphora* [119,121]. The highest aerodynamic forces in hovering, root-flapping insect-like wings are produced at an aspect ratio of approximately 3.0 [123]. As already mentioned, wing planform determines both the ability of a wing to produce lift and the span efficiency. Span efficiency for a gliding wing typically varies between 0.7 and 0.85 [106] and previous studies on animal locomotion thus used a standard generic value of 0.83 [108]. The latter value is comparatively close to the maximum efficiency of an ideal wing with elliptical shape for translation and is not reached for root flapping wings at low advance ratios. 

Flow measurements in differently-sized moths, for example, show that span efficiency in flapping flight is much smaller and varies between species. As the tested moth species had wings with similar aspect ratio and planform, there is no trend in span efficiency with increasing body size [108]. Lowest efficiency of 0.31 was measured in the smallest moth species *Hemaris fuciformis* with 0.2 g body mass, 0.6 in the intermediate-sized species *Deilephila elpenor* and with 0.85 g body mass and 0.46 in the largest species *Manduca sexta* with 1.44 g body mass [108]. These data imply that the generic value of 0.83 might not be a suitable approximation in flying insects. Eventually, butterfly wing planforms, in particular, produce elevated lift and thrust coefficients compared to any other planforms [124]. In these species, the coefficients of force production increase with increasing taper ratio and aspect ratio. This increasing performance, however, occurs at the cost of increasing power requirements for flight and thus at the cost of a reduction in aerodynamic efficiency [124].

**Figure 4:**
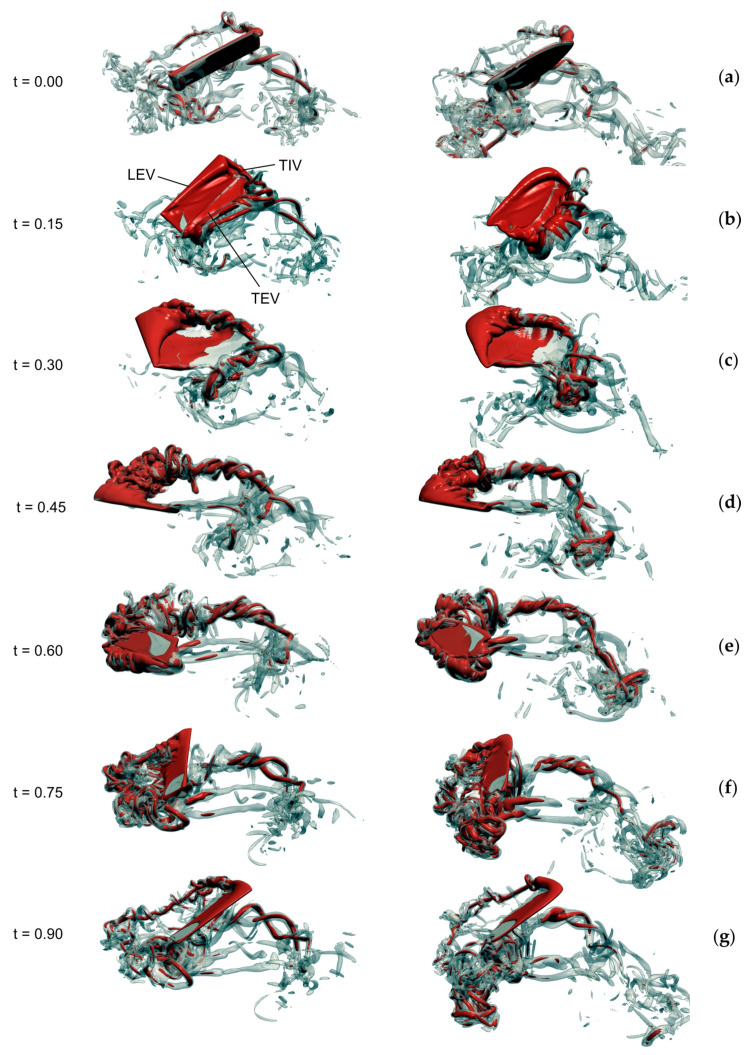
Evolution of vorticity in a flapping rectangular (left) and blowfly (right) wing. (**a**–**g**) Vorticity distribution at the beginning of the 3rd flapping cycle (t = 0–1) after motion onset. Vorticity of a flapping wing of *Calliphora vomitoria* slightly differs from the flow in the rectangular wing. Data show iso-surface with vorticity magnitude of 75 s^−1^ (semi-transparent grey) superimposed on a vorticity iso-surface with 150 s^−1^ (red). LEV, leading edge vortex; TEV, trailing edge vortex; TIV, wing tip vortex. For performance data and wing kinematics confer to Table 1 and a previously published study [83], respectively. Wing length and area are identical in both wings.

For this review, we additionally calculated the aerodynamic quantities of revolving (Figure 3) and flapping (Figure 4) wings of a blowfly, as well as simple rectangular and ideal-shaped wings in order to compare their performance. The ideal wing shape was calculated according to the estimation by Prandtl–Betz in Figure 2e. The numerical simulations were performed using a previously published numerical model [83,125] combined with a wavelet-adaptive solver [126], and efficiency was calculated as Rankine–Froude efficiency [127]. Table 1 shows that revolving rectangular and fly wings perform similarly, producing approximately the same amount of lift. The fly wing, however, produces this force at slightly higher efficiency (0.23) compared to a rectangular wing (0.22). Both values are approximately half of the values calculated from quasi-steady approach on flapping insects wings [128]. Surprisingly, an ideal-shaped wing for rotation is less effective because most wing area is concentrated at the wing base where the wing’s inflow velocity is low. The ideal-shaped wing produces ~52% less lift at ~29% less efficiency than rectangular and natural fly wings (Table 1).

Adding kinematic reversals to the revolving kinematic pattern (flapping motion) has little effect on the performance of a rectangular and natural fly wing (Table 1). However, the time evolution of lift production suggests that a rectangular wing produces more lift during up- and downstroke than the fly wing, while the fly wing produces more lift during the stroke reversals.

Although aerodynamic force production changes with changing wing planform, there is little variation in the wake behind wings with different geometry [129,130] (Figure 4). This is demonstrated by the pressure distribution of differently-shaped wings in Figure 3 and by experimental investigations on different categories of elliptic wing planforms with same aspect ratio and total area at Reynolds numbers typical for wing motion in flying insects between 160 and 3200 [130]. The latter study suggests that wake structure mainly depends on shape of the wing’s leading edge rather than planform. The authors argue that the leading edge shape determines the shear layer feeding the leading edge vortex, and thus the development of leading edge vortices and the associated flow topology [131]. Similar results are reported on mosquito flight using computational fluid mechanics and in vivo flow measurements [94]. The latter study shows that apart from leading edge vortices, also trailing edge vortices and rotational drag are responsible for elevated lift production. This was concluded from the low-pressure distribution on the suction side of the wing near the trailing wing edge. The wing planform of fruit flies, by contrast, does not produce similar low pressure regions although both insects fly at similar Reynolds numbers [94].

In general, researchers often assume that the specific wing shape of an insect species is close to an optimum, reflecting the result of a selection process on the animal’s aerial performance. A unique approach toward the aerodynamic consequences of wing planforms in flies, however, implies that wing shape also results from aerodynamically non-adaptive factors [47]. Flight tests on fruit flies with genetically modified wing shape using targeted RNA interference demonstrate that wildtype controls, with wing aspect ratios of ~2.5, have a reduced flight capacity compared to transgene animals with wings at aspect ratios between ~2.7 and ~3.0 [47]. While maximum forward flight speed does not increase with increasing aspect ratio, the transgene flies exhibit ~22% improved tangential acceleration and an ~10% improved deceleration capacity, they turned at higher angular rate (~10 – ~21%) and at an ~23% smaller turning radius than controls. The results suggest that in fruit flies, an increasing aspect ratio leads to an increase in agility and maneuverability. Notably, even if the GAL4-induced RNA interference selectively tackled wing shape, the above findings could also be explained by behavioral modifications because the maximum mechanical power output of the indirect flight muscles were thought to be similar in both tested groups [47].

## 4. Functional Relevance of Three-Dimensional Wing Shape

There is a longstanding debate on the functional relevance of three-dimensional wing shape compared to a flat wing design. It is widely accepted that the wing’s three-dimensional corrugation serves as a mechanical design element to improve stiffness and thus to avoid excessive wing deformation during flight [28,132,133,134]. Its potential contribution to aerodynamic lift and drag production, by contrast, is less clear and apparently depends on the chosen approach for analysis. The majority of previously published studies used numerical or physical wing models at various Reynolds numbers for analysis and reported that wing corrugation either improves aerodynamic performance [56,58,65,66,118,135,136,137] or attenuates performance [56,59,65,66,134,137,138,139]. Other studies that reported little or no effect of corrugation on wing performance in beetles [55], dragonflies [140], bumblebees [141], hoverflies [142], and fruit flies [50] at Reynolds numbers between 35 and 34,000. Some studies, moreover, also reported inconsistent results on the significance of wing corrugation in dragonflies [63,64,143,144], bumblebees [54], and a generic model [59].

Although corrugation may change local wing pressure, the difference of lift and drag coefficients between corrugated and flat wings is typically not more than 5% for both lift and drag for angles of attack between 35° and 50° [141], and 17% for drag at low Reynolds number of 200 and 5° angle of attack [50]. A likely explanation for the latter findings is that corrugation is usually smaller than the typical flow structures at the wing, such as the leading-edge vortex and the area of flow separation. Thus small-scale corrugation produces only small local changes in both flows at the wing and aerodynamic forces [50]. As the size of flow structures depends on Reynolds number, corrugation structures should be coarser in small insect wings than in larger wings for pronounced wing-vortex interaction. In contrast to small-scale corrugation, large-scale chordwise wing camber has a pronounced effect on aerodynamics characteristics of a wing [54,55]. Upward camber and a downward oriented leading wing edge tend to create more lift than a flat wing flapping at similar angle of attack. Chordwise camber and the shape of the leading edge are thus comparable to a change in the effective angle of attack of an insect wing [56,83].

**Figure 5:**
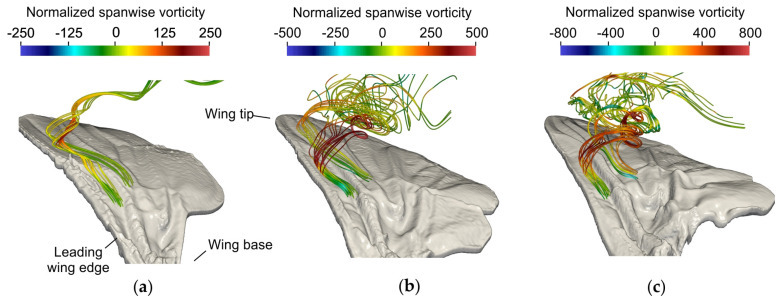
Flow pattern produced by natural wing models of three fly species. Color-coded instantaneous streamlines in (**a**) *Drosophila*, (**b**) *Musca*, and (**c**) *Calliphora*. Snapshots are taken at 1.3 (*Drosophila*) and 3.3 stroke cycle (*Musca, Calliphora*) after motion onset in natural wings [83]. Streamlines were computed from particles released in the corrugation valleys of the dorsal (upper) wing surface near the leading wing edge. Data show little spanwise vorticity inside the corrugation valley near the surface (arrows) and leading-edge vortex suction pulls the virtual particles away from the surface.

There is little difference in flow patterns between flat and three-dimensional fly wings but vortices and stagnant air cushions that are trapped in corrugation valleys of a wing may potentially improve lift production by changes in wing’s effective geometry [61,135]. Evidence for trapped vortices were experimentally found in wings moving at relatively high Reynolds number [63,64], including an aerodynamic study that demonstrated vortex trapping at the wing’s acceleration phase and at Reynolds numbers ranging from 34,000 to 10^5^, but not at 3500 [140]. The latter value is at the upper end of Reynolds numbers typical for flying insects. Studies that did not find vortex trapping attributed the absence to the elevated angle of attack in insect wings [55]. In corrugated wings of gliding dragonflies, slowly rotating vortices only develop at small angles of attack but flow broadly separates from the wing surface at larger angles (Re = 34,000 [53], Re = 1400 [136]). By contrast, a recent numerical study on root-flapping wings shows that corrugation valleys in fruit flies, house flies, and blowflies are unable to trap vortices at Reynolds numbers up to 1623 (Figure 5) [83]. Thus, small-scale corrugation, low Reynolds number, spanwise flow advecting vorticity and high angle of attack make vortex trapping less likely in flapping insect wings. Trapped flows should thus be considered as an exception rather than a common aerodynamic phenomenon in insect flight [134].

Aerodynamic studies on fly wings with genetically modified corrugation and camber patterns are missing and thus is the exact significance of wing corrugation in flies for aerodynamic performance and efficiency. This difficulty was recently circumvented by a numerical study using computational fluid dynamics on three differently-sized fly species (*Drosophila melanogaster*, *Musca domestica*, and *Calliphora vomitoria*) [83]. The wing models were reconstructed from high-resolution scans [75] and corrugation and camber numerically removed afterwards. The study allowed a direct comparison of air flow structures, force production, power requirements, and propulsion efficiency of a natural, cambered, corrugated and flat wing design. The findings suggest that three-dimensional corrugation of fly wings has no significant effect on mean aerodynamic force production compared to a flat wing at the tested Reynolds numbers for wing motion between 137 and 1623 [83]. This result is consistent with a previous study on bumblebee model wings that reported less than 5% change in aerodynamic force production of four differently-corrugated wings [141]. Our data, instead, suggest that corrugation may alter the temporal distribution of forces within the stroke cycle.

The three-dimensional camber of rigid fruit fly-, housefly-, and blowfly-wings also has no significant benefit for lift production but attenuates Rankine-Froude flight efficiency by up to ~12% compared to a flat wing [83]. This is different from previous findings on deforming wings in hoverflies, which is discussed in chapter 5 [145]. The computed flight efficiencies in rigid wings of 17–23% were somewhat below the experimentally derived estimates that range from 26–32% in various species of fruit flies to 37–55% in large crane flies, beetles and bees [23]. A potential explanation for this discrepancy is that many of the experimental studies used Ellington’s quasi-steady model for flight power [128], while the numerical model solved the Navier–Stokes equations for fluid motion. Altogether, the above results make it more likely that 3-dimensional corrugation and camber have been selected according to mechanical rather than aerodynamic constraints. Even though there are some energetic costs for wing flapping associated with three-dimensional wing shape, the increased stiffness and change in force distribution in corrugated and cambered insect wings might be of advantage during elevated wing loading—conditions that occur during maneuvering and flight under turbulent environmental conditions.

## 5. Wing Stiffness and Benefits of Elastic Wing Deformation

Wing joints, the cuticular composition of proteins and chitin fibers, and elastic proteins such as resilin allow wings to elastically deform during flapping motion in response to inertial and aerodynamic loads [24,146,147,148,149,150,151,152,153,154,155,156]. Elastic wing deformation alters flight in two ways: first, it smooths out and thus lowers sudden acceleration of local wing mass, and consequently maximum instantaneous inertial costs [116,157,158,159], and second, it changes flow conditions due to changes in local angle of attack, and thus the direction of flow [79,160]. In hoverflies, these effects appear to be negligible, as the time courses of lift, drag and aerodynamic power are similar in deforming (camber deformation, spanwise twisting) and rigid flat-plate wings [145]. Part of the potential energy stored in a deformed wing might not be elastically recycled throughout the stroke cycle, which results in plastic deformations and stress on the cuticle [161]. Moreover, the energy loss stresses the total energy budget for flight and thus leads to a reduction of propulsion efficiency. Measurements in wings of fruit flies, house flies and blowflies suggest that only 77–80% [161] and 87–93% [75] of the elastic potential energy is recycled during a full deformation–relaxing cycle. However, the significance of the relative loss in elastic potential energy depends on how much the wing deforms during flight. For example, at the end of each half stroke, aerodynamic and added mass reaction force partly cancel out wing mass-induced moments [161]. Total elastic potential energy is thus small at the end of upstroke and downstroke, and so is energy loss. Consequently, the elastic structures of the wing may not be able to recycle much kinetic energy gained from a preceding half stroke and thus contribute only slightly to the recycling of kinetic energy at the stroke reversals. By contrast, a larger amount of elastic potential energy is stored at the beginning of each half stroke and subsequently released throughout the wing translation phase in flies [161].

To avoid wing bending at elevated wing loading, spring and flexural stiffness of insect wings typically increase with increasing body size [29]. This finding also holds for fruit flies, house flies and blowflies, in which median spring stiffness along an aerodynamic characteristic beamline is ~0.024, 0.63, and 1.76 Nm^−1^, and median flexural stiffness is 4.86 × 10^−11^, 9.73 × 10^−9^, and 1.33 x 10^−7^ Nm^2^, respectively [75]. Due to these elevated stiffness values, fly wings deform only little in spanwise direction during wing flapping. Nevertheless, the distribution of local spatial stiffness in fly wings varies between species. In response to point loads at 11 characteristic points on the wing surface, for example, the average spring stiffness of bending lines between wing hinge and point load varies ~77-fold in fruit flies and ~44-fold in house flies but only ~28-fold in large blowflies [75]. This suggests that wings of larger flies behave more like a homogenous material with uniform thickness compared to smaller flies. As this property determines how inertial and aerodynamic forces deform a flapping wing, the stiffness variability could reflect the differences in local aerodynamic forces in different species.

Besides elastic energy recycling, dynamic deformations in span- and chordwise direction alter the wing’s aerodynamic performance throughout the stroke cycle [162,163,164] and may help to stabilize flight [165]. Findings on the aerodynamics of flexible wings have recently been summarized in a comprehensive review [5]. For example, Du and Sun [145] found that camber deforming and spanwise twisting wings of hoverflies produce ~10% more lift at ~17% less aerodynamic power expenditures than a flat rigid wing. The authors suggest that this benefit in lift production is mainly caused by the dynamic changes in wing camber, while the difference in power is mainly due to spanwise twist [145]. More lift at reduced costs results in an increase in flight efficiency, which in turn reduces the metabolic cost for wing flapping and may eventually enhance the animal’s fitness. Notably, this conclusion runs counter to the study on rigid fly wings that found a decrease in Rankine–Froude efficiency in cambered compared to flat wings (see chapter 4) [83]. Other examples on the significance of dynamic camber and spanwise twist include beetles and moths. Owing to force-induced deformation, wing camber in beetles is inverted (downward camber) during the upstroke that improves aerodynamic performance compared to a non-deforming wing [55]. Aerodynamic details of wings with different geometry including twist, leading edge details, and camber in hawkmoth-like revolving wings [86] show that flow separation at the leading edge prevents leading-edge suction and thus allows a simple geometric relationship between forces and angle of attack. The force coefficients in these experiments appear to be remarkably invariant against alterations in leading-edge detail, twist and camber. In general, our knowledge on the aerodynamic significance of three-dimensional wing structure and flexing in insect flight is still limited and largely stems from studies on simplified flight models such as two-dimensional computational simulations, rectangular flat wing planforms, simplified three-dimensional extrusions of two-dimensional profiles, and also from work at inappropriately large Reynolds number [54,58,59,65,118,135,136,138].

## 6. Conclusions

In conclusion, wings of insects and wings of flies (in particular) are complex, three-dimensional body appendages with elevated spanwise and comparatively little chordwise stiffness. Their tapered shape improves span efficiency during root-flapping but genetic modifications of wing shape has questioned that the current shape solely results from a evolutionary selection process towards maximum aerodynamic performance [47]. The three-dimensional corrugation pattern of veins and membranes forms valleys that channel axial flow components, following the pressure gradient from the wing hinge to the tip, but does not trap vortices for lift-enhancement as previously suggested for the more corrugated wings of dragonflies [28,61,83,135]. Fly wings also have the ability to store elastic potential energy during wing deformation, but analyses using static loadings suggest that up to ~20% of this energy might be lost due to plastic or viscoelastic deformation. Nevertheless, the exact benefits of three-dimensional wing design for locomotor capacity, flight efficiency and body posture control in insects are still under debate [166]. These data, however, are highly welcome not only by biologists working on insect flight, but also by engineers working in the area of bionic propulsion and on the development of the next generation of man-made flapping devices.

## Figures and Tables

**Table 1:** Aerodynamic characteristics of single wings with various shape during revolving and flapping motion. Wing shapes are shown in Figure 1, Figure 2 and Figure 3.

Kinematics	Property	Rectangular Wing	Ideal Wing	Fly Wing
Revolving ^1^	Vertical force (μN)	471	215	431
Revolving ^1^	P_aero_ (μW)	1696	724	1434
Revolving ^1^	Efficiency	0.22	0.16	0.23
Flapping ^2^	Vertical force (μN)	479	n.a.	458
Flapping ^2^	P_aero_ (μW)	2340	n.a.	2361
Flapping ^2^	Efficiency	0.27	n.a.	0.25

Data are calculated by a three-dimensional numerical simulation model that was refined from a previously published code (https://arxiv.org/abs/1912.05371). All tested wings have similar area (28.0 mm^2^) and length (9.76 mm), and were flat without corrugation and camber. Mean vertical force was derived from t = ~0.32 to t = 0.5 revolutions after motion onset in the revolving wing, and from the 3rd flapping cycle in flapping wings. Efficiency, Froude efficiency for wing flapping [127]; n.a., no data available. Reynolds number is calculated from mean wing tip velocity and mean wing chord. ^1^ Horizontal stroke plane, 112 Hz, 40° angle of attack, Reynolds number = 1320. ^2^ Inclined stroke plane (−20°, nose-down), 40° angle of attack during upstroke, 20° angle of attack during downstroke, 0.22 cycle for wing rotation, 150 Hz stroke frequency, Reynolds number = 1320 [83].

## Supplementary Materials

Detailed descriptions on the calculation of spanwise circulation and wing shape in revolving wings are available online at https://www.mdpi.com/2075-4450/11/8/466/s1.
